# Exploring the local policy context for reducing health inequalities in children and young people: an in depth qualitative case study of one local authority in the North of England, UK

**DOI:** 10.1186/s12889-021-10782-0

**Published:** 2021-05-10

**Authors:** Eleanor Holding, Hannah Fairbrother, Naomi Griffin, Jonathan Wistow, Katie Powell, Carolyn Summerbell

**Affiliations:** 1 School of Health and Related Research (ScHARR), The University of Sheffield, 30 Regent Court Regent Street, Sheffield, S14DA UK; 2 Health Sciences School, The University of Sheffield, Barber House Annexe, 3a Clarkehouse Road, Sheffield, S102LA UK; 3 Department of Sport and Exercise Sciences, Durham University, 42 Old Elvet, Durham, DH1 3HN UK; 4 Department of Sociology, Durham University, 32 Old Elvet, Durham, DH1 3HN UK

**Keywords:** Children and young people, Health policy, Health inequalities, Social determinants of health, Systems approaches

## Abstract

**Background:**

Improving children and young people’s (CYP) health and addressing health inequalities are international priorities. Reducing inequalities is particularly pertinent in light of the Covid-19 outbreak which has exacerbated already widening inequalities in health. This study aimed to explore understandings of inequality, the anticipated pathways for reducing inequalities among CYP and key factors affecting the development and implementation of policy to reduce inequalities among CYP at a local level.

**Methods:**

We carried out a qualitative case study of one local government region in the North of England (UK), comprising semi structured interviews (*n* = 16) with service providers with a responsibility for child health, non-participant observations of key meetings (*n* = 6 with 43 participants) where decisions around child health are made, and a local policy documentation review (*n* = 11). We employed a novel theoretical framework, drawing together different approaches to understanding policy, to guide our design and analysis.

**Results:**

Participants in our study understood inequalities in CYP health almost exclusively as socioeconomically patterned inequalities in health practices and outcomes. Strategies which participants perceived to reduce inequalities included: preventive support and early intervention, an early years/whole family focus, targeted working in local areas of high deprivation, organisational integration and whole system/place-based approaches. Despite demonstrating a commitment to a social determinants of health approach, efforts to reduce inequalities were described as thwarted by the prevalence of poverty and budget cuts which hindered the ability of local organisations to work together. Participants critiqued national policy which aimed to reduce inequalities in CYP health for failing to recognise local economic disparities and the interrelated nature of the determinants of health.

**Conclusions:**

Despite increased calls for a ‘whole systems’ approach to reducing inequalities in health, significant barriers to implementation remain. National governments need to work towards more joined up policy making, which takes into consideration regional disparities, allows for flexibility in interpretation and addresses the different and interrelated social determinants of health. Our findings have particular significance in light of Covid-19 and indicate the need for systems level policy responses and a health in all policies approach.

**Supplementary Information:**

The online version contains supplementary material available at 10.1186/s12889-021-10782-0.

## Background

### Inequalities in children and young people’s health

Improving children and young people’s health and addressing health inequalities are global policy priorities [1]. However, in both policy and practice, England can be seen to be faring poorly with outcomes for children and young people (CYP) consistently worse than those of comparable international peers [2]. In parallel we have seen increasing health inequalities between children in England for a range of outcomes including infant mortality, mental health and obesity [2, 3]. Further, child poverty is rising, with the proportion of children living in relative poverty projected to rise to 37% of children (5.2 million children) by 2021 [3]. There are concerning indications that CYP in poorer areas are being disproportionately affected by austerity measures and the greatest income losses have occurred in households with CYP [4]. This is particularly pertinent in light of the outbreak of Covid-19 which has exacerbated already widening inequalities [5] for the most vulnerable in society, such as families already living in poverty [6].

Despite agreement within the public health community about the importance of a policy focus on child health for reducing long-term inequalities in health [7, 8], there is widespread concern among child health advocates that CYP have not been prioritized in policy decision making in recent years [3, 9, 10]. Another major challenge is that current research does not address the practical uncertainties that decision makers at local levels face when trying to implement policies to improve child health at a local level. Evidence suggests that values and politics are prominent in decision-making in the local authority context, particularly as they relate to the use of evidence [11, 12]. However, Salway et al. [13] highlight that ‘our understanding of knowledge utilisation processes within the policy context is far weaker than our understanding of knowledge utilisation processes within the clinical practice environment’. While the transfer of responsibility for public health services to local government authorities in 2013 in England afforded the potential to better align the commissioning of preventative health services with existing responsibilities (for example, early years provision; planning and licensing), evidence regarding the extent of integration and alignment is mixed [14]. Local decision-makers express uncertainty and difficulty taking action in this fragmented public health system [12] and there is now an impetus to work towards developing Integrated Care Systems (ICS) to better coordinate work between the National Health Service (NHS) and local authorities in delivering public sector services [15]. The local public health system is thus increasingly characterised by restructuring, budgetary changes and integration of different organisations and priorities. It is a complex, challenging and changing context.

Developing an understanding of the local public health decision-making context, however, is pivotal if we are to understand how extremely limited resources can be deployed to promote equitable local policies that tackle the social determinants of health as they relate to CYP. Drawing on a detailed review of local documentation, non-participant observations and interviews, this study sought to explore understandings of inequality, the anticipated pathways through which health inequalities among CYP might be reduced and key factors affecting the development and implementation of policy to reduce inequalities among CYP at a local level. These key questions have relevance for and can inform understandings of and questions about local work to reduce inequalities in CYP health across the globe [16].

## Methods

### Preliminary phase: developing a theoretical framework

Alcock with May ([17]:2) describe policy as, ‘actions taken within society to develop and deliver services for people in order to meet their needs for welfare and wellbeing’. Policy stems from and cuts across a variety of mechanisms (for example, legislation, guidance, reports, service delivery etc.), levels (for example, national, regional, local, neighbourhood), and administrative and sectoral boundaries (for example, local government, Local Enterprise Partnerships (LEPs), Clinical Commissioning Groups (CCGs) etc.). Any assessment of the policy context therefore needs to take account of the historically constituted tensions in this system affecting policy making and implementation. Our approach to understanding the local policy context for reducing inequalities in CYP health focuses on three policy areas (childhood obesity, mental health and ‘best start’ (0–5 years) and the specific policies (e.g., local plans for reducing childhood obesity) that sit within these and how they interact with [22] the broader policy context (and the systems and levels that cut across these). The three policy areas were identified through a rapid review identifying national policy priorities, stakeholder engagement with practice collaborators, an investigation of priorities at a local level, identification of priority policy areas set out in the State of Child Health Report for England [3] and health and wellbeing issues with the starkest inequalities identified by the King’s Fund Report [18]. Our aim was to explore significant issues of public health equity, a diversity of issues affecting child health at different life stages and a diversity of approaches associated with health equity. We apply Jessop’s [19] notion of ‘entry points’ through using policy areas and policies to understand the role of the state and associated organisations and actors and do so by considering pathways to change in reducing inequalities in child health.

We employ a novel theoretical framework, mobilising and drawing together the work of Bacchi [20, 21] Pawson and Tilley [22] and Head [11]. First, Bacchi’s [20] ‘What’s the problem represented to be?’ policy analysis tool provides a set of questions which afford a structured means of ‘going beneath the surface’ of key policy narratives. The tool allows us to explore how inequalities are conceptualized as a necessary precursor to understanding proposed pathways for reducing inequalities. In seeking to understand how policies are expected to work, we focus on four key elements of Pawson and Tilley’s [22] realist approach: understanding the programme pathway (how is the programme understood to work); embeddedness (the extent to which the social system in which policies operate is conceptualized); open systems (how, if at all, externalities are understood and addressed in the policies) and agency (how agency is conceptualized). We also draw on Head’s [11] ‘three lenses of evidence-based policy’ formulation: Political knowledge, Scientific (research-based) knowledge and Practical implementation knowledge to explore the development of local policy. This offers a way into articulating the ‘practical craft of policy development and adjustment [...] (in which) multiple sets of evidence inform and influence policy’ ([11]:1). Through combining these approaches we developed a novel, iterative framework that unearths explicit and implicit assumptions made by participants in knowledge utilisation processes and how these relate local policy implementation targeting inequalities in CYP.

### Study design

The project utilised a qualitative case study design comprising semi structured interviews with stakeholders who worked within the field of child health across the geography of the local authority, non-participant observations of key meetings where decisions around child health are made, and a review of local policy documentation [23]. We did not specify strict eligibility criteria for potential interviewees. All staff members working within child health (such as public health practitioners, service managers, commissioners etc) were eligible for inclusion.

### Study setting

This study was based within the geography of a local authority in the North of England, UK. The specific local authority was chosen due to its high levels of deprivation (in the top 20% of the most deprived districts/unitary authorities in England) and poor health outcomes for children (for example, levels of teenage pregnancy, GCSE attainment, breastfeeding and smoking in pregnancy) compared with the England average. The case study location, therefore, provides an example of, and insights into, local policy development and implementation to reduce CYP health inequalities in a context in which health and socio-economic inequalities are reasonably high.

### Recruitment and sample

The local lead for Child Health introduced the research team to potential participants and helped to identify key meetings and relevant policy documents to review. Snowball sampling was employed to identify further participants and relevant meetings. Similarly, additional documents were identified through attendance at meetings and discussions with interviewees and following up within-policy references.

### Sample

A total of 16 interviews were undertaken between October 2019 and March 2020 with participants from Social Care (*n* = 2), the National Health Service (NHS) (*n* = 5) and the local authority (LA) (*n* = 9). Participants worked in a variety of different roles: public health (*n* = 6), clinical medicine (*n* = 5), strategy and performance (*n* = 2); education (*n* = 2) and commissioning (*n* = 1). Interviews took place at the participants’ place of work (*N* = 12) or via the telephone (*N* = 4) depending on the individuals’ preference and schedule. Six observations with 43 participants were carried out of which one was strategic (setting priorities and producing policies) and five were operational (relating to the implementation of specific policies and programmes). Each meeting was attended by a mixture of staff working within the local authority^1^ and colleagues from other organisations (such as the NHS or social care). For the documentary review, data was extracted and synthesised from both strategic (high level documents, such as wider council strategies like the Sustainability and Transformation Plan (STP)^2^ and the Joint Strategic Needs Assessment^3^ (JSNA) *n* = 5) and more operational policy documents (related to specific programmes of work (*n* = 6)). All documents, bar one PowerPoint presentation, took the form of a written report.

### Data collection methods and analysis

Interviews and non-participant observations drew on the conceptual framework outlined above [11, 20–22] to: explore the historical context for CYP policy making in the local authority area; current priorities related to CYP’s health; current decision-making processes related to CYP public health policy; expectations about how tackling health inequalities can be reduced and examples of activity related to particular local policy objectives and the experiences of implementing these (Please see additional file: Topic guide for local stakeholder interviews). With participants’ consent interviews were digitally recorded and transcribed verbatim. Observation templates were derived from the interview schedules. The documentary review extraction template also operationalised the conceptual framework outlined above. It provided opportunity for researchers to extract data directly and create memos to record ideas about what was being articulated either explicitly or implicitly within the documents.

### Analysis

Preliminary data analysis was carried out alongside data generation in order to inform subsequent fieldwork [24]. Interview transcripts and observation fieldnotes were then uploaded to NVIVo 11 software for coding. Thematic analysis adopting Clarke and Braun’s [25] systematic approach was used: once a number of transcripts and field notes were received a selection were read separately by EH, HF and KP before an initial coding framework was developed. The development and refinement of the coding framework was guided by a recurring interplay between our conceptual framework [11, 20–22] and the ‘bottom up’ method of identifying recurring patterns in the original dataset. This echoes Vaismoradi’s approach to thematic analysis [26] as consisting of both ‘description and interpretation, both inductive and deductive’ ([26], 399).

## Results

Our findings relate to three key areas: understandings of inequalities in health; perceived pathways for reducing health inequalities, and factors affecting the development and implementation of policy to reduce inequalities at a local level. The three key policy areas identified for focus in this study influenced the interview participants and documentation we selected. The findings presented are consistent across the three areas and we highlight where findings differed between interviews, observations and review work.

### Understandings of inequalities in children and young people’s health: inequality as a focus for CYP policy

When asked about their understandings of inequalities in children and young people’s health, most participants readily articulated a place focused definition, highlighting the relationship between the post-industrial nature of the town, employment opportunities, deprivation, and health inequalities. For the majority, health inequalities were understood as ‘differences’ or *‘*variation’ between specific areas within the local area and between the local area and the national average.

[national data] is the start and then it’s also just looking in-depth into the more local areas and the differences between different areas (P3).

I worked in [another area] for quite a long time, and they don’t seem to have the gaps … we’ve got, we’ve got big gaps, you know, compared to nationally (P7).

Whilst a small number of participants discussed inequalities linked to ethnicity and sexual orientation, the vast majority foregrounded socioeconomic position and inequalities between families in their descriptions. Inequality was discussed in relation to the social determinants of health within the LA, such as poverty, low employment rates and lack of opportunities, as well as prevalent health issues and practices, such as low breastfeeding rates, lack of access to services and high smoking rates. Some participants also discussed the interdependent causes and consequences of inequality, and how they impact on one another:

In a class of 30 children [in this area], there could be ten who are living in poverty and quite extreme poverty for some of them … But then, obviously the knock on effect of eating habits, exercise, being able to be in a home that’s warm, having running water. All of those things that have a knock on impact on health and wellbeing … I think there is a lot of big issues that will then have an impact on one another (P4).

Despite stating that reducing inequalities was a key priority within their work, a small number of participants acknowledged that it was difficult to provide a clear and shared understanding of the concept and aims of action at an organisational level. One participant also raised questions about responsibility and accountability in relation to equality and poverty:

We have got an equality strand. And that’s more about equality, though, than equity. So, it doesn’t feel that we’re able just to invest sufficiently in that side. And I think it’s because we don’t really have a compelling story, or we say that inequality and child health, they’re driven by child poverty, universal credit. They’re national issues. And government sort them out. And it almost allows us to wash our hands’ (P11).

Some participants cautioned against the use of the phrase ‘inequalities in CYP health’ and instead favoured discussion of ‘family inequalities’ to avoid viewing CYP health in isolation. This was particularly the case for support for children between 0 and 5 where issues affecting the whole family such as poverty or strained relationships/family breakdown were seen as inextricably linked to CYP health outcomes:

I don’t think there’s any such thing as children and young people’s health inequalities, I think there’s families and communities’ inequalities. You don’t have a child or young person living in poverty without the rest of their family being in that situation … we’re not trying to work just with the individual child or children, it’s actually the whole family... locally we can see the benefits of that working, because you can see families who don’t come back onto the radar and the system (P10).

Inequality was framed more broadly within the documentary review by focusing on socioeconomic factors as well as wider aspects of the social determinants of health. One strategic level policy document offered a broad perspective by theorising inequality as ‘unjust differences’ between ‘the social determinants of health’, which included social and economic conditions, lifestyle behaviours, ethnicity, access to services, and disability status. Other documents considered inequality in relation to the existence of ‘differences’ in poverty, deprivation and ‘social opportunities’. As with the interviews, documents tended to discuss the existence of categories of inequality (such as demographics within the local area, economic status, access to services etc) separately, rather than exploring their interrelationships and interdependences. Unlike some interview participants who acknowledged the impact of history on the area as an ex mining town, the relationship between history of place and health inequalities was largely absent from the policy documents. For example, health outcome data was predominately based on the current JSNA or other local data sources, and did not tend to explore longer term trends. Inequalities were largely understood as existing within current societal conditions, rather than being reproduced and maintained through social arrangements across history.

Priorities set out in the documentary review and statements made by participants in the fieldwork demonstrated that addressing inequalities in CYP health was an overarching focus within the LA. However, this would often be ‘unsaid’ (P7), and would not necessarily manifest itself in conversations in meetings or be ‘labelled as inequalities as such’ (P14). This was reflected in the observations where the term inequalities was only mentioned in one of the six meetings. Instead focus on inequality was framed in terms of targeted approaches to ‘reduce variation’ with particular groups and the general population, such as vulnerable families, LGBTQ young people or teenage mothers. The explicit aim to reduce inequality was more evident through the documentary review. Reducing inequality was a specific theme and key target within two strategic level documents. Operational level plans also discussed the need to focus on and reduce inequality through local work. For example, one document emphasised that planned work to reduce inequalities in early life was ‘crucial’ for improved outcomes across the life course. Both strategic and operational level plans focused on structural factors like improving the local environment, building community resilience and transforming services rather than individual behavioural factors.

I think there’s a definite culture towards [reducing inequalities], and it’s acknowledged definitely within our directorate. And I think in terms of language, [the Director of Public Health] frames everything within the language of health inequalities, and that idea of inequalities broadly as well. And that should be the central thing that we’re working towards always (P2).

Although inequality appeared to be an overarching priority for the LA, it was acknowledged that it was ‘difficult to measure improvements’ (P2) through specific measures or impact over time.

And, it’s difficult cos it’s kind of like, you know, if you are measuring inequality sort of based on deprivation I suppose is a better indicator, you know, how many families are we pulling out of poverty, rather than looking at the kind of health indicators. I don’t know. (P3).

In summary, interview participants could generally offer a definition of inequalities, which was most often framed in terms of differences in health status linked to socioeconomic position (SEP). Participants also recognised the significance of place for creating and sustaining health inequalities and linked this to deprivation and a lack of socioeconomic opportunity. Policy review work echoed this focus on SEP but also encompassed a broader reference to the different social determinants of health, including lifestyle. While interviews and observations pointed towards implicit understandings and acknowledgement of inequalities within decision-making, inequalities were more explicitly highlighted within written policies.

### Pathways for reducing CYP health inequalities: the importance of place and whole systems approaches

Throughout the interviews, participants demonstrated commitment to improving outcomes for CYP and their families. Specific interrelated and overlapping strategies to reduce inequality articulated by participants included: early help and preventative services, focusing on support within the first 1000 days of life, identifying and targeting at risk families, partnership working and organisational integration, building community resilience through interventions with families instead of individual CYP (‘whole family approaches’) and having teams based in areas of high deprivation and need (‘locality working’), rather than centralised offices. Current local priority within the LA was given to interventions which were developed from knowledge of the local area and its population, delivered within communities and tailored to local need such as the development of the Family Hubs which bring services together in order to work with families from conception to adolescence through an integrated, whole family approach. The Family Hubs are located within areas of high deprivation and need in order to build trust with local residents and ensure a comprehensive range of services are available within areas to address issues at the earliest opportunity.

I think [locality working] is about professionals building relationships in the community and being based in the community. It’s having that trusted team within the place, that people know and recognise, and can build relationships with (P15).

Similar strategies were prioritised in the documentary review, with further emphasis on the importance of consultation with CYP and families, investment in primary and community services (i.e. primary care services located within localities), taking ‘a life course approach’ (i.e. by delivering the most effective interventions for specific stages of life) and cultivating a strong public health workforce. Despite a focus on socioeconomic factors within understandings of inequality, strategies to tackle inequalities within the documents focused more on the importance of organisational integration and partnership working and less on tackling the roots causes of problems (e.g. poverty).

Discussions around the pathways to reducing inequality often led participants to reflect back on their capacity to deliver positive outcomes within the context of poverty. Some strategic and operational documents outlined the need for ‘whole system transformation’ or ‘system wide approaches’ for the reduction of poverty and the transformation of services. The need for ‘whole systems change’ to reduce health and socioeconomic inequalities was also discussed in one strategic level meeting and by two interview participants. However, in many cases the explicit meaning of systems approaches was unclear and was often discussed within the relatively narrow framing of partnership working and organisational integration.

Although child health inequalities appeared to be considered within a systems lens, there was a lack of explicit discussion of what constituted systems approaches or how to implement systems change. Instead participants’ understandings were much more implicit, revealing themselves through discussions around the importance of place, through focus on the whole family, the impact of cuts across the system, working with communities and the interrelationships between the social determinants of health. For example, one participant advocated for a ‘whole place plan’ for the reduction of obesity, which included a social determinants of health perspective that focused on the need to change the wider place and environment. However, reducing spatial inequalities of this nature was considered to be beyond the means of public health policy alone.

And we took a decision that we would move from an individualistic approach to more of a whole system approach [to tackle obesity] … And in that, we’ll have things around use of properties and things for takeaways, fast food. To try and reduce the density of fast food. But there’s not a budget call on that. What we’ve really struggled with is then saying if we wanted to go further, how might we do that as a whole place, rather than it just being childhood obesity, you say it’s a problem of public health. You better sort it. We know it’s more complicated than that*.* (P11).

In summary, participants articulated a clear commitment to reducing inequalities. They shared a variety of different strategies towards this aim (including a focus on early prevention/early intervention, whole family approaches and working in areas of high deprivation) and emphasised the importance of building on local knowledge. Similarly, review work highlighted the importance of organisational integration and consultation with local families in the development of effective policy. While reference was made to the importance of ‘whole systems approaches’ to tackling inequality, the specific ways in which this was understood and articulated varied.

### Factors affecting the development and implementation of policy to reduce inequalities in CYP health: the realities of policy implementation in the context of poverty

Participants in the interviews and observations presented a number of factors affecting the development and implementation of CYP health policy. The policy ‘problem’ was often set out early in the policy documents and related to the ‘need to do more with less’ under the ‘significant funding pressures’ of austerity Britain. This was against a backdrop of local poverty and deprivation which were frequently cited in the documents and fieldwork as key issues affecting the lives of local families (see previous theme). Described as an ‘uphill battle’ (P11), cuts to services within a climate of reduced funding and poverty led some participants to reflect on whether they were able to deliver effective services for CYP and their families. ‘Devastating cuts’ (P12) to children’s services such as Sure Start centres^4^ where family support was provided for a range of issues *‘*was a big hit for families in deprived areas’ (P12). This lack of resource often meant that public health programmes tended to target the acutely vulnerable rather than delivering preventative work to stop families reaching crisis point. Preventative work which focused on early intervention with at risk families (e.g. health visiting, Family Hubs services and the development of a toolkit for practitioners to identify early child neglect), was considered an effective mechanism for reducing CYP health inequalities by participants. However, participants were concerned over the future sustainability of preventative public health programmes. Despite clear efforts and intentions to reduce inequality through the development of early help/preventative services such as the Family Hubs, improving outcomes within this climate was perceived as extremely challenging.

There’s an awful lot of deprivation here. Some of our villages are the worst in Europe, not just in the UK. Austerity has hit [this area] very hard. Cuts have been quite deep. A lot of our children’s centres in areas that really needed them have closed … a lot of those early help preventative services closed … you have to spread themselves very thinly and concentrate on the most vulnerable, rather than doing as much preventative work as they would like to do. So, it’s impacted across the whole system really. Those little cuts in every area, when you put them all together, have had a devastating effect (P13).

Although poverty was considered the most pressing factor leading to inequalities in health within the area, there was less discussion about how local public health policy could contribute to a reduction in poverty. One participant felt this lack of attention was due to local and national policy often being too focused on one singular issue (such as education legislation), rather than collective action to mitigate ‘the root causes’ (P4) of problems. This participant criticised national policy’s failure to recognise the interrelated and overlapping social determinants of health. Policies focused on improving specific health outcomes for children were seen as futile if not aligned with policies impacting upon the wider determinants of health.

I think one thing exacerbates another, and some of the problems we have, for me, for national policy, is some policies are singularly focused, rather than focusing on root cause of problems... I think my call to the national government, is that there needs to be much more joined-up policy working at Whitehall. If you’re going to put policies out about health inequalities, for example in children, that’s got to take account of the DWP’s policies around income and Universal Credit and things like that. (P4).

In the same vein, participants emphasised the importance of national policy to reduce poverty as a prerequisite to any local, health outcome specific policies. The impact of poverty on efforts to reduce inequalities meant that some participants spoke of targeting resources at services which are not focused on underlying causal processes, such as a tooth brushing scheme as described by P11. Their account demonstrates the limitations of some services in the face of extreme poverty. It also shows how downstream, behavioural interventions can be a distraction of focus and resource away from the upstream efforts to tackle structural inequalities.

Where we’ve had opportunities, we’ve tried to do that. We’ve tried to get the healthiness team, the school nursing services, to target more of their resource at the more deprived, more needy areas. I think that it’s a combination of the dose that we’re giving isn’t quite enough. And some of that probably just reflects the reality of life for a lot of people in this area. If 30% of our kids are living in households that are in poverty, the supervised tooth brushing scheme is probably not going to offset the impact of child poverty. So, one of my reflections would be even if you got a good programme locally and well resourced, if your local situation is such that your wider determinants are going against you, you’re really fighting an uphill battle (P11).

Restrictions around funding also posed problems for effective joint working in the delivery of services. Although many participants reiterated how strong partnerships had been developed across organisations within the LA, others reflected on the difficulty of delivering integrated services whilst *‘*fighting for contracts’ (P15). According to a small number of the participants working within the NHS, commissioning of services can lead to organisations withholding information from other providers because they do not wish to lose their competitive advantage when seeking future funding. This finding potentially undermines the delivery of effective ‘joint working’ and ‘integrated services’ across local CYP systems, which was identified by participants and in statements in the policy documents as important for improving CYP outcomes. It also demonstrates the challenges involved in partnership working to reduce health inequalities within local areas.

I do think there are things that should be happening that aren’t happening [to reduce inequality]. And I think some of that is around competitive tendering and competitive contracts. I think that is a huge barrier to services working together because services become really precious about their information and about the way that they work … people are so terrified of losing parts of their service. (P12).

Effective joint working was also challenging due to what one participant labelled as the *‘*public health paradox’(P3); which they described as conflict of interests between public health professionals and colleagues in other LA departments interested in increasing footfall and business to the area (such as the opening of new fast food restaurants). Their account demonstrates understandings of systems thinking which links organisational, social and economic priorities.

For me, I think there’s still a lot of silo working so, you know, like we just sort of talked about sort of people addressing issues in their kind of, in their silo and not really thinking about, you know, determinants or other things that might be impacting on that individual or that family or community … I suppose that links into the whole systems working where you’ve got conflicting priorities, particularly within the local authorities … and it, it really does stop you making progress in some respects. (P3).

As already described, many participants focused on structural factors leading to health inequalities by reflecting on the poor quality of the local built environment and the lack of exercise facilities, green space and abundance of hot food takeaways and its impact on health outcomes. However, within discussions of the challenging environment of the local area a small number of participants drew on individualised accounts of the attitudes and culture of local people. Participants reflected on how negative health behaviours had become ‘accepted norms’ (P5) perpetuated within families and used this to help explain the lack of success of public health programmes.

I’m not sure we ever get any outcomes, positive outcomes from all the work that happens … Do we get any small wins, do we get any? No cos it’s just getting worse. Society is getting worse. [This area] is just getting worse, and I think that’s a real challenge … I think it’s cultural*.* (P5).

Our interview data also revealed a perceived disconnect between national policy development and local policy implementation to reduce CYP health inequalities. Despite a number of participants discussing the merits of national policy for ‘setting direction’ (P3), pulling together evidence in a time of reduced resources, as well as demonstrating a statutory function to help make the case for future investment, many felt that national policy was ‘unrealistic’ (P10), ‘idealistic’ (P13) and ‘dissociated with reality’ (P8). Participants drew on an in-depth knowledge of the local area to describe the difficulty in implementing nationally developed programmes within their local context. Using the example of the Stronger Families initiative^5^; a national programme which assists families with complex circumstances through a whole family’s approach, one participant described how the specification and targets ‘did not match’ (P12) local families. This led to ‘missed opportunities’ which ‘could have saved decades of inequalities’ (P12). Despite great efforts from those involved in the delivery of the intervention, the specific targets set by national government in this case left little flexibility for using local knowledge to assess eligibility for inclusion.

Stronger Families...We did it wrong. But actually the concept the idea was right but we focused on specific targets that have to be met. We actually lost sight of what we should have been doing …. that made us not be able to really hit the families we should have been hitting the ones that we all knew that might not tick those boxes because people might be in work at the moment. But actually, these families didn’t quite hit those criteria, but we know these families experience decades and decades of inequality that we could have probably then changed some lives. (P12).

Others discussed how national policy tended to be detached from the realities of life in the North of England by failing to take account of regional and economic disparities. This meant that policy did not always adequately recognise the interrelated nature of the social determinants of health and how these relate to spatial inequalities of place.

What isn’t always helpful is when policy seems a bit distanced from reality, not necessarily linked. It’s a bit generic for local places. London is very much different to [this area], and sometimes it feels a bit disassociated from reality. (P8).

Participants described how the disconnect between national policy discourse and local policy processes led them to adapt and tailor policy to local need. Despite reference to the need for policies and interventions to be evidenced based, published academic research was rarely mentioned in the development or implementation of policy. Instead participants used their individual knowledge (based on experience acquired over years of working in the same place) alongside locally collected data to shape national guidance, particularly when policies were attached to national funding. Others chose to disregard national policy they considered irrelevant to their local context to focus on areas of greater need. For some, national policies, such as Future In Mind,^6^ which offered greater flexibility and room for local interpretation, were seen as the most effective for improving outcomes.

When policy comes down and you receive it, and then layer it over your local intelligence and work out, that would shape your thinking. But obviously when there’s funding attached to it and mandate, then well actually, do we need that money for that particular area, when we know we’ve got greater need elsewhere? I just think that sometimes it’s easier to write a policy, the art is in interpreting it and implementing it locally (P8).

Throughout the fieldwork and review work it was evident that local action to reduce inequalities was perceived to be extremely challenging in the face of high levels of deprivation and cuts to local budgets. National policy was highlighted as an important mechanism for setting direction but was criticised for focusing on discreet policy areas rather than whole systems, a lack of sensitivity to local contextual characteristics and not doing enough to tackle the wider determinants of health.

## Discussion

Drawing on a detailed review of local documentation, non-participant observations and interviews with key stakeholders, this study sought to explore understandings of inequalities, the anticipated pathways through which health inequalities among CYP might be reduced, as well as key factors affecting the development and implementation of policy to reduce CYP inequalities at a local level.

Given the high levels of poverty within our case study site, it was not surprising that most participants invariably framed health inequality as variation in health outcomes due to socioeconomic inequality. Defining inequality as differences in outcomes between populations and social groups corresponds with several definitions of health inequalities within the literature [27]. Further, focusing on variation in socioeconomic factors over other aspects of the social determinants of health, including the impact of social relations (such as racism and sexism) on health outcomes, corresponds with international literature on health professionals’ understandings of inequality (see, for example, [28]). A key feature of health inequalities definitions in the literature is that these differences and variations in outcomes are unjust unnecessary and avoidable [27]. Although participants did not explicitly acknowledge the unfair nature of inequalities in their definitions, it was clear that inequalities were a key focus of their work. However, as that some participants struggled to articulate a clear and shared definition of their understanding of inequalities highlights a need to develop and agree understandings at an organisational level.

As well as participants understandings about inequality our findings highlight a number of pathways targeting reductions in CYP inequality such as early help and preventative services (such as health visiting and the Family Hubs), identifying and targeting at risk families, locality working in areas of high deprivation, focus on the whole family and place based/systems approaches. However, our study also highlights the impact of poverty and austerity, alongside concern about, for example, the implications of commissioning of services and targets for the prioritisation of local systems around reducing CYP health inequalities.

Our findings reinforce the importance of place for understanding differences in health and social outcomes. Participants understood and recognised the significance of the social determinants of health and how these relate to spatial inequalities between and within places. For example, participants understood inequality as variations in outcomes both within the local area and between the local and the national. In foregrounding socioeconomic differences within areas, i.e. pockets of deprivation, and their impact on inequalities in health outcomes, participants underscored the significance of local context for shaping health and illness trajectories. Similarly, although reference to history of the local area was largely absent from the documentary review, many participants discussed the history of the area as an ex-industrial town and its impact on inequalities, deprivation and a lack of social and economic opportunities. This links with an established body of literature on the effect of place on geographical differences in health and illness [29]. For example, Macintyre et al. [30] highlight social, economic and physical environments exert both direct (e.g. pollution) and indirect (e.g. access to services) influences on area level health. Engaging with context of place is integral for understanding differences in health outcomes which ‘may be in part of a legacy of past experiences, socio-economic conditions and demography’ ([31], 478).

Our research also highlighted the cumulative impact of austerity across local services and on the ability of local systems to reduce CYP health inequalities. This reinforces findings from other research which shows the uneven impact of austerity according to geography. Cuts to budgets and the impact of welfare reform have disproportionately affected vulnerable populations in areas of high deprivation and need [32, 33]. Indeed, participants in this study reflected on the impact of austerity across the local system but within specific pockets of need, such as areas of high deprivation where cuts to services such as Sure Start centres were most acute.

In recognising the importance of place and its impact on spatial inequalities participants also recognise how action to reduce health inequalities form part of a systems approach. Understanding socioeconomic trajectories and the local service delivery context is important in the implementation of systems approaches to reducing health inequalities [34]. There are growing calls within public health to move away from a dependence on lifestyle factors to approaches which acknowledge the complex and interrelated factors that generate inequalities in health [34–37]. Taking a place-based, community-centred approach acknowledges that geographical areas do not experience health inequalities equally – and that contextual factors such as demographics, leadership and socioeconomic conditions may have a wide range of effects on outcomes [34]. However, such an approach requires that services work together around common goals to address CYP inequalities [2, 8, 38] with effective action across all levels of government, as well the NHS, the third and private sectors and community groups [7, 38].

Despite a number of participants and policy documents advocating for systems approaches to reduce inequalities in this study, our findings highlight the very real challenges of taking a joined-up approach within the competitive structures of contract commissioning and in conditions of austerity. Some practitioners demonstrated a good understanding of how systems function, such as the importance of linking of organisational, social and economic priorities to address the social determinants of health. However, while a whole systems approach to tackling the root causes of inequality is identified as a priority, the current political economy in which local governments operate obstructs this [39]. For example, reduced budgets and competition lead some providers to withhold information that may assist other organisations in attracting further funding. This chimes with previous research which shows how commissioning and contracting structures in health and care services creates competition and fragmentation in policy development, service delivery and governance. It can be extremely difficult to build trust and share information within competitive commissioning processes [40]. Prioritisation of different outcomes and silos working presents challenges for partnership working within public health [41]. Similar to our findings, low trust relations may also encourage opportunistic behaviour such as the sharing or withholding of information at the disbenefit of another party [40].

In the case of this study, the likelihood of commissioning structures bringing organisations together to make local solutions is somewhat limited given the intractability of regional socioeconomic conditions, budget cuts and cuts to services. As with Powell et al. [42], despite engagement with the social determinants of health, these institutional structures led some participants to focus back on individualised understandings of health (such as toothbrushing) rather than the root causes of problems, which Lorenc et al. [43], argue are unlikely to have a substantial impact. By failing to acknowledge such externalities in the local policy making process, national policies are not truly *embedded* within the social and economic systems that (re-)produce the social determinants of health [22]. This disconnect between national policy development and local policy implementation led participants to utilise their contextualised understanding of the local area (or practical implementation knowledge [11]) to engage in a process of funnelling and tailoring of national policy to local need and place (e.g. development of whole place plan, focus on the whole family and locality working in areas of high deprivation).

The prevalence of poverty and deprivation were discussed as having a particular impact on the lives of local families and the ability of local systems to reduce inequalities in our case study site. The link between child poverty and negative health, social and educational outcomes is well-established and longstanding [38, 44], with recent research highlighting the impact of poverty on child health and widening inequalities due to austerity in the UK [2, 3, 45]. Our participants consistently highlighted the limitations of local service delivery in the face of poverty, echoing previous research in the UK [41] and the US [28] where public health professionals have articulated a perceived lack of capacity to impact upon the social determinants of health at a local level. It is clear that reducing poverty and deprivation are largely beyond the means of local policy makers alone. Although some policy levers exist at a local level (such as changes to the local environment) the ability of local policy makers to effectively use them is heavily dependent on decisions made at a national level [29]. A whole systems approach which acknowledges how the social determinants of health manifest in place and are a part of local systems is required to reduce poverty and inequality [34]. However, according to our participants and wider literature, there remains a lack of joined-up policy attention to reduce poverty at a national level [2, 45]. Local and national policy were seen by some participants as too narrowly focused (e.g. on one policy area) to address the root causes of problems (such as poverty) through collective action. National policy was seen to be too idealistic, failing to recognise regional and economic disparities, and lacking attention to the interrelated nature of the social determinants of health and how these interact with, and are constrained by, contextual characteristics of place.

It is important to highlight, however, that a focus on reducing poverty alone (within our study and reflected in the wider literature) may not narrow inequalities in child health. Reducing health inequalities is particularly challenging because they are a moving target requiring areas with the worst health to improve at a greater rate than those with best. In this respect, Scambler and Scambler [46] have argued that the mechanisms giving rise to and sustaining differential access to wealth and power need critiquing, given their role in shaping socio-economic inequalities in society and the pattern of health and health care. Rowlingson [47] and Lynch [48] have raised the significance of redistribution of wealth via increased taxation or labour market regulation for narrowing health inequalities. However, we also need to acknowledge that tackling inequality through such policy intervention may be politically unappealing given public support for redistribution in the UK has been relatively weak [47, 49]. In this respect, focusing on poverty rather than redistribution (as is the case in this study) for example, leaves those at the ‘top’ of inequalities unproblematic and may also inadvertently stigmatise families living in poverty as ‘the problem’ [20, 21].

Given the increased focus on the importance of systems approaches at a national level and the introduction of Integrated Care Systems (ICS) in the UK as a means of bringing together NHS and local councils in healthcare delivery [10], our study has drawn important attention to the challenges faced by policy makers and how these interact with socioeconomic trajectories at a local level. Our findings both support previous literature and add to this as they emerged in an era where systems approaches are increasingly advocated as a means of reducing health inequalities, but show that organisational barriers to implementation remain and, crucially, interact with the wider socio-economic characteristics of place. It is clear that participants in this study are engaging with systems approaches and the social determinants of health for the reduction of CYP health inequalities at a local level. However, our findings also suggest that local efforts to address the social determinants of health require proportionate universal resourcing from national government in order to reduce inequalities. Whilst disparities in regional socioeconomic resources remain, local service providers do not consider a systems approach will be able to reduce CYP health inequalities alone.

Our findings carry particular significance in light of the Covid-19 pandemic as they provide understanding of the nature of system level effects and highlight the barriers associated with, and the importance of, taking a systems approach to reducing inequalities. Current policy responses to the pandemic in the UK have been piecemeal and ‘patchwork’, such as education support through the (delayed and disjointed) delivery of laptops for the most disadvantaged [50]. A number of international policy responses have been introduced as a means of alleviating the effects of CYP poverty, such as temporary suspension for housing evictions (e.g. Spain, France, some areas of Canada), or child care support for parents working in essential services (e.g. Austria, Italy, France and the Netherlands) [51]. Such approaches are reactive and do not address the underlying causes of poverty (such as the distribution of resources) at a systems level. Our study points towards the need for a health in all policies approach which aims to improve health by embedding health decision making across organisations, sectors and policy areas [52].

### Strengths and limitations

A key strength of our study is the creation of a novel, iterative framework, which mobilises and draws together the work of Bacchi [20, 21], Pawson and Tilley [22] and Head [11] to guide the development of our data collection materials and interpretation of the results. Further, we employed three different data collection methods (interviews, observations and a documentary review) affording an in-depth insight into the challenges faced by local policy makers in the development of CYP policy. Such attention is significant given increased calls for policy responding to CYP inequality [9]. As an in-depth case study of just one local authority in the North of England, however, there is an important limitation given that the findings may not be replicated in the same way in other settings with different trajectories, levels of deprivation and population demographics. However, local authorities across England share many common challenges with significant cuts to budgets and rising poverty across the country, exacerbated by the outbreak of Covid-19. It is important to recognise that providing a detailed understanding of work to reduce inequality in one local authority area can offer theoretical generalisability [16] in the sense that a contextualised exploration of one setting can inform understandings and questions about others. Indeed through focusing on a local area with high levels of both socioeconomic and health inequalities our study has resonance for work across the globe to reduce inequalities in CYP health at a local level.

## Conclusion and recommendations

This study explored participants understandings of inequality, the pathways to reducing inequalities and the key factors affecting the development and implementation of CYP health policy at a local level. Our findings highlight a number of anticipated pathways to reducing CYP inequality such as early help and preventative services, identifying and targeting at risk families, locality working in areas of high deprivation, focus on the whole family and place based/systems approaches. Despite notable challenges in the delivery of services many participants demonstrated a commitment to maintaining a social determinants of health approach to policy. This was demonstrated through understandings around the importance of place, systems change, whole family approaches and the interrelationships between categories of inequality.

Despite awareness of the social determinants of health, efforts to reduce inequalities are thwarted by the prevalence of poverty, reduced funding and cuts to vital public services. It is significant that these barriers remain despite repeated calls for and work towards taking a ‘whole systems approach’ to improving public health and reducing inequalities [34, 37] There is a clear need for more joined up policy working at a national level which takes into consideration regional disparities, allowsfor flexibility in interpretation, and involves actors across the system to address the different and interrelated social determinants of health. This is particularly pertinent inlight of Covid-19 which has brought already widening inequalities in health in the UK [5] and other countries such as the US, to the fore [53]. The pandemic has alreadydisproportionately impacted the most vulnerable groups in society, particularly families already living in poverty [6]. Given that improving children and young people’shealth and addressing health inequalities are global policy priorities [1], there is a pressing need for national policies to focus even more on lifting families out of povertyand working to redistribute resources in order to reduce inequalities in child health.

Like Marmot et al., [38] our study draws attention to the challenge of targeting the social determinants of health and reducing inequality at a local level in the context of austerity and in the absence of an overarching national health inequalities strategy. Recommendations from Marmot et al., [38] provide a way forward for localities to take actions to tackle health inequalities in this challenging context, whilst calling for a national strategy for action on the social determinants of health within a framework of a proportionate universal allocation of resources to target inequalities. Local policy makers should look to develop a health system which considers inequalities as unjust and puts a commitment to reducing health inequalities at the core of local action. Key to this will be the development of a consistent definition of health inequalities at an organisational level that should be understood and agreed by all partners.

Local policy makers should continue to shift focus from downstream, behavioural interventions and invest in public health programmes focused on prevention and early intervention (such as the Family Hubs in our case study site). Such services should be based within communities and tailored to local need and place. Further, as acknowledged in this study, the impact of interventions to reduce inequalities are difficult to measure and quantify within short time frames. Organisations should be less tied to traditional measures of success and be enabled to use knowledge of their specific local context to develop a more innovative understanding of what success looks like over time. Finally, and most importantly, local policy makers, alongside other child health advocates, should continue to press for investment and a commitment to reducing inequalities in child health at a national level.

## Supplementary Information

**Additional file 1.** Topic guide for local stakeholder interviews.

## Data Availability

The qualitative datasets generated and analysed during the current study are not publicly available due to participant confidentiality and anonymity that form part of the ethical approval of the project, but are available from the corresponding author on reasonable request.

## Abbreviations

CYP: Children and Young People
LA: Local Authority
ICS: Integrated Case Systems
NHS: National Health Service
LEPs: Local Enterprise Partnerships
CCGs: Clinical Commissioning Groups
STP: Sustainability and Transformation Plan
JSNA: Joint Strategic Needs Assessment
LGBTQ: Lesbian Gay Bi Transgender Queer

## Footnotes

1 Staff working within the local authority refers to individuals who work within local government in a specific locality.

2 Sustainability and Transformation Partnerships are new partnerships between the NHS and local authorities which are designed to deliver services on a more coordinated, system wide level

3 Joint Strategic Needs Assessments is a process by which local authorities and CCGs assess the health needs of their populations through analysis of local intelligence to inform decision making.

4 Sure Start children’s centres is a programme which targets children under four living in disadvantaged areas. They are community hubs which are designed to provide help and advice on child and family health, money management, family relationships, training etc. Since 2010 more than 500 centres have closed.

5 The Stronger Families initiative targets families with complex issues through a coordinated, whole family approach. Each family receives support from one key worker in order to build their resilience, stop them reaching crisis point and reduce demand on services

6 Future in Mind is a government initiative designed to transform CYP mental health services
