# Supplementary material for: Exploring the local policy context for reducing health inequalities in children and young people: an in depth qualitative case study of one local authority in the North of England, UK
Source: BMC Public Health. 2021 May 10;21:887. doi: 10.1186/s12889-021-10782-0 (PMC8107408; doi:10.1186/s12889-021-10782-0)
Supplement: Supplementary file 1 — Additional file 1. Topic guide for local stakeholder interviews. [file 12889_2021_10782_MOESM1_ESM.docx]

**SPHR CYP WP1: Topic guide local stakeholders**

**Introduction:**

- Thank you for participating; introduce self, SPHR & study
- Key points: your perspectives on facilitators and barriers of developing and implementing policy to reduce inequalities in child health, specific focus on 3 policy areas
- No right or wrong answers & participation is voluntary, rights to withdraw
- recording interview (concentrate on what you are saying, accuracy)
- Confidentiality and anonymity
- Free to clarify / correct anything said at any point in the interview
- Questions?
- Happy to proceed? Sign consent form

START RECORDING

**Participant background/role**

- Organisation (purpose; main activities) - remit for child health
- Clarify current role and responsibilities, particularly in relation to child health

**Local context**

- What you perceive to be the current priorities for child health in this LA?
- What do you perceive to be the key inequalities in CYP in this LA?
- What are the main drivers of inequality in this local area?

**Priority setting**

- What local evidence is drawn on in setting priorities?
- How are priorities agreed?
- Have priorities changed in recent times / remained largely the same?

**Policy development process**

- What is the process for developing policy in relation to key priorities? Who has responsibility for taking the decision about priorities forward?
- Who is involved in developing local policy relevant to child health? (community, children, parents, third sector?) Extent of involvement
- Anyone who could / should be involved but isn’t currently?
- To what extent do the targets set monitor reduction in health inequalities?
- How / to what extent is local action to reduce inequalities incentivised / rewarded?

**What is the role of national drivers in influencing local policy?**

- How do you work out which national policies to work with?
- Who is responsible for keeping up to speed with national drivers/ policy?
- How useful is national policy - strategic / is it practical e.g. use of tools / frameworks – if not why not? What is missing? What would you like to see?
- How does national level documentation compliment / add to (or not) local knowledge?
- Are proposals for the implementation of national policy at a local level adequate for your local context?
- If national policies do not complement local knowledge, how does this affect the policy process?
- We’re focusing on 3 policy areas: obesity, mental health and best start.

1. **In relation to these policy areas, can you tell me about:**

- Whether they are local priorities
- If yes – how did they become a priority? (drivers: evidence, national policy/guidance)
- If not – why not?
- What is going on locally to improve outcomes

1. **To what extent is inequality a focus of this work?**

- Are inequalities measured?
- Do initiatives differ for different groups / areas?
- Do policies take a more individual or structural approach to targeting inequalities? Or both?

1. **What is working well in relation to reducing inequality?**

- What facilitates productive work to reduce inequality?

1. **What is not working so well?**

- What are the barriers to reducing inequality? Are these barriers specific to the local authority/policy area/or particular demographic groups?
- What could be improved?
- What should be happening to reduce inequalities?

1. **What national drivers or particular policies are relevant?**

- How have they been used?

1. **Have the community been involved in relation to policy work in these areas**?
2. **Thank and close**

- Ask if anything else they'd like to add
- Any suggestions for other participants to take part (and can they facilitate introduction)
- Any key documentation that would be useful to share with study team
- Thank and close
